# mHealth and telemedicine utility in the monitoring of allergic diseases

**DOI:** 10.3389/falgy.2022.919746

**Published:** 2022-09-02

**Authors:** Violeta Kvedarienė, Paulina Burzdikaitė, Inga Česnavičiūtė

**Affiliations:** ^1^ Institute of Biomedical Sciences, Faculty of Medicine, Vilnius University, Vilnius, Lithuania; ^2^ Faculty of Medicine, Vilnius University, Vilnius, Lithuania

**Keywords:** mHealth, mobile applications, COVID-19, allergy care, mobile health, telemedicine, monitoring of allergic diseases, teledermatology

## Abstract

This literature review discusses the use of mHealth technologies and telemedicine for monitoring various allergic diseases both in everyday life and in the context of COVID-19. Telemedicine, whose popularity, and demand has skyrocketed during the pandemic, rely on mHealth technologies, video calls and websites as a resource-saving and safe way of consulting patients. The incorporation of new mHealth technologies into telemedicine practice may not only be relevant in the context of pandemic restrictions but can also be applied in everyday medical practice as an effective method of patient counseling. The mobile healthcare applications include a wide range of mobile apps for patients' education, monitoring, and disease management. However, applications for the people with food allergies lack relevant information about allergies and, like most other applications, are developed without the contribution of healthcare specialists. During the COVID-19 pandemic, low-risk food-allergic patients were able to rely on telemedicine services where they could get the help, they needed without increasing risk of contracting COVID-19 while saving time. Meanwhile, some applications for allergic rhinitis and asthma patients are showing practical benefits in clinical trials by allowing an efficient assessment of treatment regimens and efficacy. The use of digital symptom diaries further facilitates the implementation of real-life studies. However, for respiratory allergic diseases, the often insufficient quality of pollen prediction needs to be taken into account. Even though studies have shown that asthma is better controlled with mHealth technologies, the quality of mobile apps for asthma patients varies widely, as many products provide information that has not been scientifically proven. Inhaler sensors – have been shown to improve the course of asthma and its monitoring, while push notifications prompting people to take their medication double the likelihood of treatment adherence. Teledermatology has a high level of patient satisfaction – as it is perceived as a more time-saving method of consultation. However, the diagnostic accuracy of contact consultations remains higher. mHealth technologies provide a patient's health data from his/her daily life, which enables insights into behavioral patterns. This closer look at the daily routine can have a significant impact on developing individualized treatment and care guidelines.

## Introduction

With the inexorable growth of the impact of mobile technologies in healthcare, the World Health Organization (WHO), together with the International Telecommunication Unit (ITU), has established the “Be Healthy, Be Mobile” initiative (1). It promotes the development of mobile health (mHealth) in the national health systems to help fight diabetes, cancer, cardiovascular and chronic respiratory diseases. The WHO defines mHealth as the field of public and medical practice related to mobile devices (mobile phones, tablets, and other wireless devices) (2). In addition, in May 2021 the Medical Device Regulation of the European Parliament and of the Council has entered into force, which focuses on patients' health and the quality of medical devices. The aim of the adapted regulation is to fix quality standards for software and mobile applications in the healthcare sector. Therefore, the number of low-quality apps is expected to decrease with the end of the transition phase in 2024 (3).

According to recent publications from the European Academy of Allergy and Clinical Immunology (EAACI) (4) and the American Academy of Allergy, Asthma, and Immunology (AAAAI) (5), mHealth can be useful in providing high-quality medical care to patients with various allergies especially those with allergic rhinitis (AR) and asthma (6). mHealth has great potential to improve the quality of healthcare, expand access to services, reduce costs and improve personal and public health (7). As the COVID-19 pandemic has challenged healthcare systems and medical practices around the world, the need to communicate with patients remotely has emerged (8). The benefits of telemedicine technologies have been observed in ambulatory and home-care settings, such as the “Covid-Guide” initiative developed by the German Central Institute for Statutory Health Insurance Physicians during the COVID-19 pandemic, which allows patients to self-assess their health complaints, alerts them to the possible association of their symptoms with COVID-19 infection, and identifies and educates them on the key COVID-19 symptoms (9). And in an example that is closer to the present day - in the face of the COVID-19 pandemic, a Telemedicine Consultation System (ETCS) was established by the National Telemedicine Center of China (NTCC), located in Zhengzhou, Henan Province, which has virus outbreak alert and response network system and has benefited in patient monitoring and multidisciplinary care (10). According to a 2020 German study of 2,827 medical staff respondents, the importance of telemedicine and teleconsultation during the COVID-19 pandemic was rated as high or very high by the majority of respondents (doctors (1036/1806, 57.4%), nurses (508/797, 63.8%), and other medical professionals (90/127, 70.9%)) (9). However, only 20.2% of university hospitals, 20.3% of private clinics and 5.6% of regional clinics routinely used teleconsultation in clinical practice. In contrast, 36% of physicians in private practice used telemedicine routinely in their work (9).

Another survey-based German study, conducted in 2020, interviewed 71 allergists. 46.5% claimed to have used telemedicine applications before the start of the COVID-19 pandemic, and 72.3% said they used telemedicine technology after 31 January 2020. When comparing the frequency of use of teleconsultation methods before and after the pandemic restrictions, video consultations jumped the most in popularity (4.3% vs. 15.6%) (11). Mobile health applications (or mHealth) are increasingly being used to support telemedicine. Advances in audio, video and data telecommunication technologies have made it easier for doctors to communicate with patients remotely (12). Telemedicine using mHealth devices and applications could be an accessible, accurate and cost-effective complement to face-to-face visits taking into account individual patient cases and situations (13). However, it also has its limitations. The development of a mobile application is not difficult technically, does not require a very high initial investment or extensive knowledge of the disease itself. According to 2017 data, 318,000 mHealth apps existed and 200 more were being developed every day (14). Most of these apps have not been tested on patients and are not approved by recognized health regulatory organizations such as the Food and Drug Administration (FDA) in the United States of America (USA) or the European Medicines Agency (EMA). Patients and healthcare professionals will be increasingly encouraged to use apps that have not been tested for quality, safety, efficacy, reliability, and appropriateness by any public health authority or scientific organization. The inappropriate use of apps and other telemedicine tools can jeopardize the continuity of the patient-doctor relationship by increasing the tendency for the patient to monitor his/her treatment without consulting specialists.

This article reviews currently available mobile apps for diagnosis, monitoring, and treatment adjustment of major allergic diseases, their clinical and practical utility, reliability, and limitations, as well as the use and benefits in the context of the COVID-19 pandemic.

## Methods

Scientific publications were searched in the databases “Academic search complete (EBSCO)”, “Medline”, “PubMed”, “Science Direct”, “Web of Science”. The following keywords were used in the search: e-medicine, mobile health, mHealth, telemedicine, mobile application, allergy, allergic disease, COVID-19. Publications selected for analysis according to the criteria: (1) publication in English; (2) published in 2004–2022; (3) the content examines the practical, clinical utility, reliability or drawbacks of current mobile applications designed for the diagnosis, monitoring, or treatment of the major allergic diseases. Data were analyzed using descriptive analysis.

### Current state and impact of mobile health and telemedicine

Mobile healthcare applications include a wide range of mobile apps for patients' education, monitoring, treatment, diagnosis, and prevention. Most of these apps can be downloaded to mobile devices from the Apple Store or Google Play, some of them are free of charge. Based on “Statista” reports, 84.7% (6.567 billion) of the world's population owns a smartphone today, and it is estimated to reach 7.690 owners by the year 2027 (15). In the US study that collected data from 2017 to 2018 National Cancer Institute Health Information National Trends Survey (N = 6789), 85.22% of respondents had a smartphone, 46.83% reported having a health app on one of these devices, 43.23% reported the use of their smartphone or tablet to track progress on a health-related goal and finally, 34.66% reported that they have a device other than a smartphone or tablet to track their health or behaviors (16). As stated by 2022 “Grand view research” data, the growth of digital market is estimated at a compound annual growth rate of 27.7% from 2022 to the year 2030, reaching USD 1.5 trillion by 2030 (17). mHealth is a promising area for the healthcare system and the main factors driving the growth of this market are the increasing integration of digital healthcare into practice and advances in digital technologies (17).

Telemedicine is the remote consultation of patients for diagnosis, treatment and disease tracking based on telecommunication technologies. Telemedicine includes video and audio calls, online sharing of photos, video or voice recordings, emails, and text messages. The main objective is to improve access to medical care. Telemedicine was introduced more actively during the COVID-19 pandemic, but given the demand from consumers, the continuous advances in technology and the drive to make medical practice more efficient, it is likely that telemedicine will continue to be used more and more in the future. The main areas of development are chronic disease monitoring and patient care in remote (e.g., rural) areas.

### Mobile apps and for monitoring allergic rhinitis

Allergic rhinitis monitoring using various apps has been in use for several years. For example, “Allergymonitor” allows tracking of symptoms and medication use, which are also compared to local pollen concentrations (18–20). A 2014 study conducted in Germany showed that patients who used the “Allergymonitor” app were more adherent to their medication regimen than the control group and improved their knowledge of the disease (21). Another symptom and medication diary is “Husteblume”, which further includes location-specific pollen forecasting, individual correlation of symptoms and allergen exposure as well as information on treatment options, triggers and disease mechanisms (22). A prospective German study evaluated 143 users of the “Husteblume” app: 55.9% of the patients reported that they learned more about their disease by using the app, 27.3% reported an improved quality of life, 33.6% had a better management of their disease, 28.0% felt better prepared for a medical consultation by using the app, and lastly, 90.9% did not identify any adverse effects of the app (22).

Another widely used app, “MASK-air®”, is available in 28 countries and has been translated into 21 languages (23). The app uses lists of medications that are adapted to each country and a visual analog scale (VAS) to assess allergy control (general allergy effects, symptoms of rhinitis, conjunctivitis, asthma), sleep, and work productivity (24, 25). Patients fill the medication intake and VAS in the application daily and may even receive a reminder message for that. The app also includes pollen season and air quality forecasts and additional questionnaires such as CARAT, EQ-5D-5l and WPAI:AS (26). Users of the app learn more about the nature of their symptoms, track increases in pollen concentrations and can therefore monitor their illness more effectively (26).

In an Australian study that aimed to identify an effective app for the self-management of allergic rhinitis and/or asthma, the most effective of the 418 apps selected was found to be “MASK-air”, which scored an average of 0.91/1 MARS points (27). “MASK-air's” daily recording of medication use and VAS allows for a more accurate assessment of treatment efficacy. This is a more objective assessment of the treatment of allergic rhinitis than patient complaints, as allergic rhinitis is known to be a variable disease and control is highly dependent on allergen exposure. Studies with “MASK-air” confirm that patients rarely adhere to the recommended treatment regimen, discontinue treatment when they feel better and consume more medications when symptoms worsen (28–31). It has also been shown that patients who take multiple medicines have poorer disease control than those who take only one or no medicine at all (30). Only less than 5% take their medication according to their doctor's recommendations (30). Treatment guidelines assume that patients adhere to their treatment regimen, but clearly better patient education and involvement in the development of the treatment plan is needed (26). Also, studies with the “MASK-air” application have found a statistically significant correlation between VAS scores for work productivity and VAS scores for allergy control (32–35). This may be significant when assessing the cost-effectiveness of different treatments (26).

The application may be useful in allergen immunotherapy (AIT). AIT is the only one that can alter the course of allergic disease, as the administration of a dosed allergen alters the immune status of allergic rhinitis. As the diagnosis and treatment is complex and takes years, the patient's well-being is prioritized. The latter is assessed by means of a visual analog scale. A symptom-drug scale is also used to assess the effectiveness of AIT. The EAACI has proposed a combined symptom and medication score (CSMS) for the assessment of AIT (36), but studies with the “MASK-air” have shown that it does not correlate sufficiently with VAS estimates of work productivity (r = 0.56; *P* < 0.0001) (37). Therefore, a new CSMS with a higher correlation (r = 0.82) was developed (37). It is planned that using this CSMS in combination with geolocalized patient pollen and air pollution data, it will be possible to compare daily exposure to allergens and pollution with the CSMS [Allergic Rhinitis and its Impact on Asthma (ARIA)-EAACI-CSMS] (37). Using ARIA-EAACI-CSMS, the clinician will be able to assess the daily adherence to the treatment regimen, the need for medications, the severity of allergic rhinitis and asthma control, as well as the impact on work productivity after the first pollen season (37). This will allow the selection of patients requiring AIT and it should improve the efficiency of AIT and be cost-effective (37). Using ARIA-EAACI-CSMS, the clinician will be able to assess for each AIT patient the exact symptoms and medications used during the pollen peak and compare them to the previous year (37). This will improve overall decision-making and determine whether AIT is worth continuing (37). It is crucial for the COVID-19 situation when direct contact with the treating doctor is difficult. Currently, AIT is offered for a period of 3–5 years and is stopped without an objective assessment of its effectiveness (31). This approach allows the clinician to decide more objectively after 3 years whether to offer to continue or discontinue AIT and, the patient will be monitored after discontinuation of AIT, which will allow to assess whether treatment should be reinitiated (37).

A study analyzing 9 mobile apps providing pollen forecasts in Vienna (Austria), Berlin (Germany), Basel (Switzerland) and London (UK) showed that the quality of the forecasts is insufficient (38). This is because the pollen season does not necessarily correlate with each patient's symptoms (39), and even sub-micron pollen particles can cause severe symptoms such as thunderstorm asthma (where breathlessness attacks increase before a thunderstorm) (40). Furthermore, the definition of the pollen season is not yet fully clear (41), although efforts are being made to define it as precisely as possible (38). There is often a weak correlation between pollen concentrations and symptoms (42). However, study, conducted in Tasmania, Australia, characterized non-linear associations between airborne pollen counts and respiratory symptoms reported by 2,272 users of the “AirRater” app between the year 2014 and 2019 (43). The study found a non-linear relationship between total pollen counts and respiratory symptoms up to three days after exposure and the association was strongest on the day of exposure and synergistic with particulate air pollution (43). Pollutants and weather conditions can interact with pollen to cause allergic rhinitis and asthma symptoms (44). Patients with AR should avoid more polluted roads when walking, cycling, or exercising. In many cities, traffic air pollution concentrations decrease rapidly within a few hundred meters of roads, and apps (e.g., “Cycleevancouver”) can help people find alternative routes (45). Another study was conducted in two Australian cities, Melbourne, and Canberra, between 2014 and 2016 - during the grass pollen seasons - by collecting airborne samples of grass pollen and smartphone AR survey responses (>96,000 submissions) (46). The study showed that the variables most useful in predicting AR symptom scores were whether the user took the medication, daily pollen concentration, time of day (46). The study also showed that pollen, weather conditions, particulate matter and demographic data can help to predict daily AR symptoms, crowd-sourced AR symptom data can be used to predict current grass pollen levels, and in-app symptom reports can be used to generate personalized AR symptom predictions (46). mHealth technologies, such as mobile apps (e.g., AirRater), not only allow patients to record their daily symptoms, but also make it possible to collect a large number of user-reported symptoms at a given location and time (43). In the future, symptoms monitoring combined with the assessment of individual exposure (indoors and outdoors) is expected to play an important role (47).

### Mobile technologies for bronchial asthma patients

More than 500 mobile phone apps have been developed for asthma patients (48). A 2017 systematic review of the literature that included 12 randomized controlled trials demonstrated improved asthma control with these apps, even though the quality of the apps was generally quite variable (49).

Most asthma apps have been developed for adults, but some have also been developed for school-age children and adolescents (50–54). In addition to reminding people to take their medication on time, pop-up messages can also use Global Positioning System (GPS) to remind them to take the medication every time they leave home (e.g., “Asthma”). Patients can fill an asthma diary on the app – symptoms, impact on quality of life, medication use, spirometry data and send messages to their doctor (e.g., “Asthma manager”). Some apps are even connected to spirometers (e.g., “SpiroHome”) or inhalers (e.g., “BreatheSmart System”) that send data directly to the app. Also, by filling in symptoms and treatment data in the apps (e.g., “Breathe”), it is possible to get an assessment of disease control and advice on how to improve it (e.g., encourage avoidance of provoking factors). In addition, the apps provide step-by-step information on what to do in the event of a severe asthma attack (e.g., “Asthma”). The doses of the medicines taken can be summarized and a notification can be given that it is time to refill stocks.

Randomized controlled trials comparing the effectiveness of mobile apps and paper diaries in controlling asthma have shown that there is no significant difference in asthma outcomes between paper-based tools and applications users (54, 55). However, adolescents' satisfaction with mobile applications was found to be extremely high, with as many as 100% saying they would recommend the app to a friend (54). Other studies focus more on the benefits of mobile programs in improving adherence to the treatment regimen. For example, a recent randomized controlled trial involving 42 patients developed an application with several features, the most important of which was to improve adolescents' adherence to their medication through regular questionnaires and pop-up messages (56). The results showed that the application significantly improved medication use (56). A multicenter study of the European Union-funded “Horizon 2020” project “My Air Coach” to develop an innovative asthma monitoring system has recently been completed (56). The “MyAirCoach” system consisted of an inhaler sensor, an indoor air quality monitor, a physical activity meter, a portable spirometer, a nitric oxide fraction exhalation device and a mobile app with asthma questionnaires, graphical instrument results, outdoor air pollution meters and other features (56). The study showed that the system was clinically effective in improving asthma control, exacerbation rates and quality of life and the users of the mHealth platform reported positive evaluations of the system (56). Additionally, systematic literature review including two apps and 7 clinical trials, showed that the use of the apps integrated with asthma inhaler sensors improved adherence to the treatment regimen and reduced the use of short-acting inhalers but did not affect the asthma control test score (57).

A 2015 systematic literature review of 147 apps reported that 13% of existing asthma apps made recommendations on disease monitoring that were not supported by scientific evidence (58). Non-evidence-based apps used as medical tools can be harmful. Since the development and promotion of mHealth apps do not require evidence that they improve asthma outcomes, it is difficult for patients and healthcare providers to choose appropriate and effective applications for their own needs.

Some applications used for the monitoring of allergic rhinitis is also suitable for use in patients with bronchial asthma (e.g., “MASK-air”). The application can be used not only for daily control of the disease, but also to monitor the need for medication. The collected data is useful for the patient to assess his own disease control and adherence to the treatment regimen as well as for the treating physician, as it remains in the application and the doctor can see and track for his workspace.

### Mobile technologies for allergic skin diseases

mHealth can play an important role in the treatment of patients with dermatological allergic diseases such as atopic dermatitis, contact dermatitis, chronic urticaria. Once a diagnosis is confirmed, applications can be useful to track symptoms, encourage adherence to treatment regimens, facilitate doctor-patient communication, engage patients in support groups and conduct clinical trials.

Apps can assess the severity and spread of the disease by keeping a diary of the disease and recording the use of medication (e.g., “Eczema Manager”). The apps use validated questionnaires to keep statistics on disease progression, treatment effectiveness and other aspects. The Patient-Oriented Score of Atopic Dermatitis (PO-Scorad), the Atopic Dermatitis Activity Score and the Patient-Oriented Eczema Measure of the University of Nottingham have been validated for the assessment of the severity of dermatological diseases and have been adapted for use in mobile apps (59, 60). In addition, apps have been developed that run on smartwatches and can record a patient's night-time scratching (e.g., “Itch Tracker”) and sleep quality (e.g., “Fitbit sleep tracker”). These apps allow a more objective assessment of symptoms and their impact on sleep.

Mobile applications that provide information about the disease, treatment, playful information for children, videos and patient stories can promote self-management of the disease, as patient education improves adherence to treatment regimens (58). Inappropriate or inadequate use of medication and treatment failure is more likely to occur due to a lack of patient knowledge. For patients with allergic skin diseases, apps have also been developed to remind them to take their medication on time and to adhere to their treatment regimen (60).

Clearly positive or negative skin test results can be photographed and evaluated by morphometric analysis using software such as Adobe Photoshop® (61). A 2007 study showed that digital morphometric analysis is an accurate and objective method of assessing skin prick test response and can be used regardless of the patient's skin color (61). Automatic image recognition can also provide additional support to professionals. However, no application or algorithm has yet been proposed for practical use.

### Mobile technologies for food allergic patients

In 2015, 77 food allergy-related mobile apps were analyzed in a US study (62). Some of the apps only provide educational information (24.6%), but the majority (67.5%) also offer a variety of practical tools such as food ingredients readers (27.5%), food (23.5%) and symptoms (21.5%) tracking diaries (62). Only 6 of the 77 apps contained both educational information and practical tools (62). Other useful features found were restaurants locators for patients with food allergies and educational games for children (62). However, none of the apps allowed to create a personalized food allergy action plan by the physician (62). The authors concluded that most of the apps lacked relevant information on food allergies and were developed without the involvement of healthcare professionals (62).

To help consumers identify allergens in foods, the 2014 European legislation [Regulation (EC) No 1169/2011 on the provision of food information to consumers] requires businesses to clearly indicate to consumers on labels or other verbal or written communications information on the nutritional value of the product/meal (whether packaged or unpackaged) and the presence of any of the 14 specified food allergens (cereals containing gluten, crustaceans, eggs, fish, peanuts, soya, milk, tree nuts, celery, mustard, sesame, sulfur dioxide, lupins and clams) (63). The barcodes and QR codes used for food labeling can be scanned by apps (e.g., “ShopWell®”, “ipiit®”, etc.) that indicate whether a product contains one of these common food allergens and can even suggest alternatives (4). Applications to identify food allergens are widespread but unproven and often do not declare the source of information (4). Other apps (e.g., “Alerje”) help allergic patients to choose the right food based on the patient's specific allergen profile, i.e., by entering the names of the allergenic food substances, a barcode can be scanned to see if the product contains the allergenic ingredients highlighted in the allergy profile (64). However, mobile apps cannot be relied on completely, as food may be contaminated with allergenic food substances during cooking or processing (4). Therefore, the authors of a position paper declare that, effective warning systems would be extremely useful (4). For patients traveling to countries where their mother language is not spoken, apps (e.g., “Bon Apetit”) have been developed to translate food names into pictures or other languages (4).

A systematic literature review conducted in 2022 evaluated 48 apps for allergen identification, support for gluten allergies, recipes for individuals with food allergies, health education, allergy support or communication, allergy-friendly restaurants, help with travel (e.g., translating food allergies or ingredients and locating allergy-friendly hotels), storing allergy profiles, allergy diary (64). Quality assessment using MARS (Mobile App Rating Scale) showed that the overall quality score of the 48 apps is acceptable, based on MARS mean ratings of 3.31 ± 0.43 out of a maximum of 5 points (64). The apps with the highest average ratings were for the categories of recipes, allergy profiles, allergen identification, and health education. Interestingly, the highest rated apps on the MARS were not downloaded more frequently, with a poor correlation between app quality and user ratings (64). Mobile health can have a significant impact on the diagnosis and prevention of food allergy, but it is important to assess the clinical relevance of these apps before recommending them to patients to avoid over diagnosis and adverse reactions due to inaccurate information. Thus, close collaboration and further research between different stakeholders is essential in the development of applications for food-allergic patients.

### Anaphylaxis, educational and preventive mobile apps

Currently, mHealth apps are commonly used for the education of patients with anaphylactic reactions (65, 66). Recognizing and dealing with anaphylactic shock is not only important for patients, but also for family members, teachers, preschool staff, nurses, and others who may encounter with it (4).

These apps include pictures and videos that teach how to recognize an anaphylactic reaction. An anaphylaxis care plan with audio instructions and an automated emergency call. The epinephrine injector can be connected to a mobile app (e.g., “Rescufy”) to alert when the user is too far from the injector. New alert systems are currently being developed to identify and locate people with epinephrine autoinjectors in the vicinity and the nearest emergency departments, but these apps still lack the full evaluation (4), monitoring time, patient and public education to be widely used. Automated alerts signaling to the patient that their epinephrine auto-injector has run out have already been successfully used (67, 68). A randomized controlled trial involving 100 subjects in 2018 showed higher patient satisfaction with the smart case for epinephrine autoinjectors compared with conventional autoinjectors [60% and 80%, respectively, (*p* < 0.05)], with a significant reduction in anxiety in those using the device (69). In addition, participants reported more frequent carrying of the injector (69), which results in faster medical aid to themselves or relatives in case of anaphylaxis.

### Mobile medication reminders

mHealth can be useful to monitor patients’ adherence to treatment regimens, to identify the underlying factors, and to encourage adherence. Many mHealth apps exist to remind patients to take medication on time. A 2016 systematic literature review and meta-analysis including 16 randomized controlled trials showed that mobile phone notifications double the likelihood of taking medication, with a 17.8% increase in adherence to regimen (70). However, a systematic literature review in the same year, which evaluated 272 mobile apps to remind people to take their medication, found that most apps lacked sufficient features and/or were of poor quality (71). Thus, mobile technology notification-reminders, patient self-monitoring apps are a promising strategy to improve the use of smart-technology-based health care in the management of both allergic rhinitis and other chronic diseases.

### Allergy telecare during the COVID-19 pandemic

Coronavirus disease, caused by severe acute respiratory syndrome coronavirus 2 (SARS-CoV-2), was identified for the first time in December 2019 in Wuhan, China. The disease has spread rapidly and has swept across the globe (72). To control the spread of the pandemic and protect individuals, urgent adjustments have been made to the health care system, including the reduction of face-to-face visits and the integration of telemedicine into practice. Allergy practices were no exception to the new guidelines and regulations (73). In a survey conducted in Turkey in 2020, 183 allergists responded that they used telemedicine tools for asthma (73%), allergic rhinitis (53%), atopic dermatitis (51%), chronic urticaria/angioedema (59%), drug hypersensitivity (45%), food allergy (48%), allergy to insect venom (30%), anaphylaxis (22%) and hereditary angioedema (28%) (74). Although well adapted, telemedicine does not overshadow the importance of face-to-face visits for more serious allergic diseases such as anaphylaxis or hereditary angioedema (74).

During the COVID-19 pandemic face-to-face evaluation for allergic rhinitis patients can be postponed or can be implemented by remote health care tools, also can be transferred to telemedicine visits for initiating or monitoring the treatment (unless there are circumstances that require an urgent visit to an allergist) (75). Not only was/is healthcare more difficult to access during the pandemic, but during the flowering season, patients with allergic rhinitis can confuse their symptoms with a COVID-19 infection (76). While the mentioned mobile technologies can track the condition of an allergic rhinitis patient, it is important for clinicians to differentiate allergic rhinitis symptoms from other conditions and adapt the care accordingly (76).

Individuals with asthma are particularly susceptible to COVID-19, and a steady treatment regimen, appropriate use of the medication and optimization of the course of the disease are all important in easing the condition (76). Mobile devices can be used to evaluate spirometry data of patients with asthma. This may include peak expiratory flow devices, portable electronic spirometers, portable exhaled nitric oxide monitors and new digital health tools such as smartphone microphone spirometers (77). Such mHealth technologies facilitate telemedicine by improving remote monitoring and management of a patient's condition through patient data (78).

Recent studies have shown that patients with asthma who receive telemedicine counseling have similar health outcomes to those who receive face-to-face monitoring (79). Furthermore, there is no current evidence to suggest that biologic therapy for asthma increases the risk of infection during COVID-19, and therefore the current international guidelines recommend the continued use of biologic therapy for the treatment of asthma during COVID-19 (80). During a pandemic, it is advisable to postpone visits for patients with mild, moderate or well-controlled asthma, for whom telemedicine technology can be used to ensure monitoring of their condition and adequate supplies of medicines (75).

To combat the spread of COVID-19, strict measures were taken to restrict the flow of people, to distribute care to high-risk patients (emergency visits, surgical procedures), and to meet the need for non-emergency consultations with the use of teledermatology services. The use of and demand for teledermatology services has increased during the pandemic (81). There are three types of teledermatology services: synchronous (real-time *via* video call), asynchronous (patient data is sent online to a physician, who reviews the information and responds few minutes or days later), and hybrid (the two latter technologies combined) (82).

The benefits of asynchronous dermoscopic teledermatology were highlighted in the 2020 survey - physicians were provided with a dermoscopic image in addition to the macro image, which increased the accuracy of diagnosis from 45.3% to 53.6% (83). A 2020 study in China looked at the role of teledermatology in the context of COVID-19, data from 698 patients were collected (82). The study showed that people aged between 20 and 39 years were more likely to use teledermatology services due to the increased use of smartphones, and the most common condition for which patients required telemedicine care was eczema (82). One of the mobile apps used for teleconsultations was “WhatsApp” (82). As it is not possible to examine the skin lesion with a dermoscope or palpation during the teleconsultation, patients should pay attention to the quality of the pictures sent, as this is the only way for the physician to establish the diagnosis (82). In the USA the leading app both before and during the pandemic remained “Teladoc”, which, according to the vendor, offers the possibility to get a dermatological consultation for conditions such as eczema, psoriasis, rashes, acne, aging and skincare (84). The 2020 analysis did not reveal how many people have used telemedicine specifically for dermatological problems, but it is estimated that many people have used telemedicine for skin conditions - in April 2020, dermatological conditions were the fifth most common condition diagnosed during telemedicine consultations in the US (85).

Repeated visits to the doctor are known to lead to a better disease control (86). A 2017 systematic review of the literature including 21 clinical trials found that the diagnostic accuracy of contact consultations is higher compared to teledermatology in the diagnosis of skin cancer, but some studies show a relatively high diagnostic accuracy of remote consultations (87). Teledermatology has also been found to reduce waiting times for consultations, and patients are more satisfied and willing to pay for the service themselves (87). The function of messaging with health care specialists offered by various websites allow prompt answers to simple questions and thus avoids unnecessary contact consultations (87). It has been concluded that telemedicine is a cost-effective and time-saving method of patient follow-up, especially for patients whose access to doctors is complicated (87). In 2017, a survey in the Netherlands involving 99 healthcare professionals and 9 patients found that the biggest benefits of telemedicine are the ability to contact doctors online, request for prescription renewals, share photos, view medical records, and consult a doctor more frequently (88). Reduced consultation time and privacy concerns have been identified as the main disadvantages of telemedicine (88). A prospective study conducted in 2016 in the US with 300 patients compared telemedicine with face-to-face consultations (89). The average waiting time was reduced from 114 days to 39 days for patients who had a teleconsultation (89). Those in the telemedicine group also paid 14% less (89). Although studies show high overall satisfaction with telemedicine, in 2017, a randomized controlled trial conducted in the USA found that both patients and dermatologists reported a significantly higher preference for a contact consultation (*P* = 0.001) (89). As mentioned before, the ability to consult patients remotely is a particular advantage in the current COVID-19 pandemic.

Allergists faced many challenges during the COVID-19 pandemic, but new paradigms of medical care may help overcome the difficulties. One innovation is the use of telemedicine to lower-risk food allergy procedures especially for patients living in rural areas (90). The allocation scheme for allergy and clinical immunology services was drawn up in 2020 by a consensus-based *ad hoc* group of experts from the United States of America and Canada (75). According to the scheme low-risk food allergy procedures where virtual health could be considered during and after COVID-19 include virtually supervised early allergen introduction in infants (for example infants with mild-to-moderate eczema, infants with an older sibling with peanut allergy), virtually supervised oral food challenges (for example patients with an unconvincing history of food allergy in combination with negative or weakly positive skin prick and/or sIgE testing) and virtually supervised oral immunotherapy (AIT) (for example peanut AIT for lower-risk patients; AIT counseling/education before initiation of AIT; AIT follow-up to assess adherence) (75). The use of such telemedicine services helps to ensure stable access to healthcare services while reducing the risk of contracting COVID-19 (75).

During the COVID-19 pandemic, delays in emergency access, long waiting times in admissions, and the possibility of infection with COVID-19 has led to a rethinking of anaphylaxis management tools and options (91). In the Academic Allergy Unit in Milan, Italy, which was previously the epicenter of the COVID-19 infection, only high-risk patients, such as asthmatics, were provided with ongoing care in an outpatient setting (92). However, patients at risk of anaphylaxis were provided with contact consultations in cases of treatment with nebulized agents, administration of maintenance doses of immunotherapy, diagnostic oral food challenges (92). Other visits (such as administration of biologics other than omalizumab) were accomplished by telemedicine (92). According to this approach, management of anaphylaxis should be limited to home injection of epinephrine, unless the patient has a history of extremely severe, near-fatal anaphylaxis (92). Telemedicine support during a pandemic would ensure adequate prevention and, if necessary, treatment of anaphylactic reactions (92).

Telemedicine can play an important role in allergy care and can transform the current models of care (93). A retrospective study in the UK looked at 439 non-face-to-face patient telemedicine visits for outpatient consultations in a tertiary adult allergy center during the second month of the pandemic - the overall experience of telemedicine services was very good/good for most patients (85%) (93). Although telemedicine cannot fully replace face-to-face visits, its application during crises such as pandemics is an important tool in clinical practice, but it is important to consider the individual case of each patient.

## Conclusions

Telemedicine and mHealth technologies are a promising and growing field. Although these technologies will not take the place of face-to-face visits, several studies have already demonstrated their effective role in clinical practice in the day-to-day and pandemic settings. Allergy practice is no exception - mHealth technologies and telemedicine can be widely used (e.g., management of allergic rhinitis and asthma, allergic skin diseases, food allergy, anaphylaxis, etc.).

The clinical and practical relevance of mobile applications developed for the management of allergic rhinitis and asthma has been demonstrated in clinical trials. The latter apps allow patients to track changes in pollen levels and take preventive measures accordingly. It also allows doctors to monitor patients' adherence to the treatment, monitor the response to medications and adjust treatment. mHealth apps combined with asthma inhaler sensors have also proven to be useful in practice, improving adherence to the treatment and reducing the use of short-acting bronchodilator inhalers. Furthermore, the range of mobile applications for food allergy patients is very broad.

Mobile applications can not only make patients' daily routine easier, but also help to prevent adverse allergic reactions. However, there is still a lack of studies demonstrating their reliability and applicability in practice. Moreover, mobile phone messages that remind patients to take their medication on time are a very promising tool to encourage adherence, increasing the likelihood of medication adherence by as much as two times.

Telemedicine, an area of particular interest during the COVID-19 pandemic, is a cost-effective and time-saving method of monitoring and treating patients. Incorporating telemedicine into medical practice is an effective way to remotely monitor patients' health status, educate them, and prescribe medication when it is not possible to do so in person, for example in the context of pandemic constraints. However, telemedicine consultations also have drawbacks, such as the inability to carry out some medical interventions. This could lead to inaccurate diagnoses or improper treatment. On the other hand, telemedicine, which has become particularly popular during the pandemic, can also be used in everyday life, saving resources and time.

Mobile tools are useful for anaphylaxis prevention, with smart epinephrine injectors holders experiencing less anxiety and more frequent carrying of the injector. There are prospects for mHealth technologies and telemedicine - e.g., mHealth technologies for allergic rhinitis and asthma are expected to be used to stratify patients for allergen immunotherapy and to standardize indications for discontinuing treatment while the integration of telemedicine into everyday practice is also welcomed.

Thus, while mHealth and telemedicine face many challenges it is a promising part of the diagnosis, monitoring, and prevention of allergic diseases. Although this area of healthcare is still in its infancy, with close collaboration among healthcare and information technology professionals and public education. It is expected to play an important role in improving access to medical care and monitoring of chronic allergic diseases.

eHealth technologies have certain limitation such as lack of clinical trials, the need for standardized rules, privacy, security, licensing, and language barriers. Some recent articles may have been missed in our review due to differences of spelling keywords or quickly development of technologies in medical area.
